# Anesthetic Management of a Neurosurgical Patient With Amyotrophic Lateral Sclerosis: A Case Report

**DOI:** 10.7759/cureus.64492

**Published:** 2024-07-13

**Authors:** Bhanupreet Kaur, Navneh Samagh, Amit Narang, Shashank Paliwal

**Affiliations:** 1 Anesthesiology, Adesh Medical College, Bathinda, IND; 2 Anesthesiology, All India Institute of Medical Sciences, Bathinda, IND; 3 Neurosurgery, All India Institute of Medical Sciences, Bathinda, IND

**Keywords:** riluzole, scalp block, chronic subdural hematoma (csdh), anaesthesia, amyotrophic lateral sclerosis

## Abstract

Amyotrophic lateral sclerosis (ALS) is a progressive form of neurological disorder that affects both the upper and lower motor neurons. Anesthesia management in these patients is always challenging as they can develop respiratory complications because of pre-existing muscle involvement. We report a middle-aged male with ALS posted for chronic subdural hematoma evacuation (CSDH) surgery. Surgery was done under scalp block with monitored anesthesia care. The choice of anesthesia in these patients should be one that interferes the least with the disease pattern while still providing optimal conditions for surgery.

## Introduction

Amyotrophic lateral sclerosis (ALS) is the most common motor neuron disease that affects both upper and lower motor neurons (LMNs) in the brain and spinal cord. It is a progressive neurodegenerative disorder that causes motor neuron degeneration and death. There is muscle denervation, which involves voluntary muscles and respiratory muscles [1], leading to voluntary muscle weakness and respiratory failure. The etiology of ALS is not known. However, many sporadic and genetic possibilities are suggested. It presents with a diverse clinical heterogeneity [2]. Initially, it presents with signs of LMN degeneration that usually affects the upper extremity, but it can also present with an upper motor neuron (UMN) or bulbar symptoms. There is no cure for ALS; medications can only help to reduce symptoms and improve their quality of life. The global incidence is 1-2.6 per 1,00,000, with a prevalence of four to five per 100,000 [3]. The mean age of involvement is 64 [4], and the lifetime risk of developing ALS is 1:400 for women and 1:350 for men [3]. There are four stages of ALS: early stage, middle stage, late stage, and end stage. Our patient was in middle-stage ALS. These patients suffer from hampered mobility due to muscular weakness and require assistance in day-to-day life. They are frequently prone to falls. We report a case of a 28-year-old male with ALS who developed chronic subdural hematoma (CSDH) due to a fall and was posted for CSDH evacuation surgery. Due to muscular weakness, he was bedridden, and anesthesia was planned to minimally hamper the disease. Surgical interventions requiring (general/regional) anesthesia may accelerate the progression of ALS due to the direct influence of anesthetic drugs, inflammation, and hypoperfusion. Burr hole evacuation was done in scalp block with monitored anesthesia care (MAC). General anesthesia (GA) carries the risk of aspiration/ventilatory depression, hyperkalemia, and rhabdomyolysis. The regional central neuraxial block may exacerbate the preexisting neurological disease. GA with fentanyl and propofol or total intravenous anesthesia (TIVA) using volatile is considered to be safe. Among the regional anesthesia, peripheral blocks appear to be safe [5]. Moreover, regional blocks can prevent perioperative surgical pain and reduce the need for analgesic medication with corresponding side effects. Thus, it was performed in the regional block with a backup of GA.

## Case presentation

A 28-year-old male with a known case of ALS for 10 years was diagnosed with right fronto-temporo-parietal CSDH and posted for burr hole and evacuation. There was a history of falling from bed 15 days back when attendants noticed a progressive decrease in the consciousness of the patient. The patient was brought to the emergency, and a non-contrast-enhanced computed tomography (NCCT) scan was done, which revealed a right fronto-temporo-parietal acute on chronic subdural hematoma. The patient was scheduled for burr hole evacuation.

A pre-anesthetic evaluation was done. The patient had a known case of ALS in the middle stage with no other comorbid illness. His disease started 10 years ago. The patient had a history of slow onset and progressive weakness, which started from the right fingertips and progressed to the full body. On examination, at present, the Glasgow Coma Scale (GCS) was E2V1M6 with bilaterally equal and reactive pupils. Motor power was 1/5 in all limbs, with spasticity in both upper and lower limbs. The patient had motor aphasia, dysarthria, and an inability to ingest solid food. Bowel and bladder incontinence was present. The sensory system and cranial nerve examinations were normal. Superficial reflexes were intact. Deep tendon reflexes could not be elicited. Cognition was intact. Eye muscles were spared. He was bedridden for the last five years and required wheelchair assistance for movements. He was on glutamate inhibitors (tab Riluzole 50 mg twice daily) for the last five years regularly and was on regular follow-ups for the disease in a tertiary care hospital.

Blood investigations, chest X-ray, and electrocardiography results were normal. Airway assessment showed mouth opening of three finger breadths, modified Mallampati (MMP) grade 3 and normal thyromental and sternomental distances. Pulmonary function tests could not be done due to decreased consciousness of the patient and emergent nature of surgery. It was planned to do surgery under MAC and scalp block. Informed consent was obtained prior to the procedure, and he was fasted to solids for six hours and to water for two hours. No premedication was given.

On the day of surgery, the patient was carefully shifted to the operating table, and the supine position was supported with the help of pillows due to spasticity in the limbs. All standard American Society of Anesthesiologists (ASA) monitors were attached. Baseline vitals were normal. Room air saturation was 95%. Although the planned anesthesia was a scalp block with MAC, a backup of GA was kept ready, which included oral and nasal airway, appropriate-sized laryngeal mask airway (LMA), endotracheal tubes, video and direct laryngoscope, drugs like propofol and fentanyl, and inhalational agents like sevoflurane. The maximum safe dose of local anesthetics was calculated. Under strict aseptic precautions, bilateral scalp block was given with 2 mL of 0.5% bupivacaine at each nerve site, i.e., supraorbital, supratrochlear, auriculotemporal, zygomaticotemporal, greater auricular, greater occipital, and lesser occipital nerves [2]. Oxygen is supplemented via nasal prongs at 2 L/ minute. After painting and draping, an incision was made at the highest point of the hematoma, and no change in vitals was observed. Surgery was continued. Intravenous paracetamol was given for intraoperative analgesia. Intraoperative vitals and EtCO_2_ were monitored. The duration of the surgery was 45 minutes. The patient was shifted to the intensive care unit for the first 48 hours and then to the ward and was finally discharged after five days. The patient was discharged with GCS of E4V1M6 and was advised to follow up after 15 days.

## Discussion

Anesthesia management in ALS is challenging, especially in patients requiring neurosurgical procedures. A high index of suspicion is required as neurological signs are masked by the pre-existing neurological deficits due to ALS. The symptoms of ALS include generalized limb weakness, bulbar muscle weakness (1%-9%), and respiratory muscle weakness (1%-3%) and may present with head drop or truncal muscle weakness [1].

Quantification of traumatic brain injury is difficult as GCS assessment is tricky in the background of profound neuromuscular weakness. The choice of treatment for CSDH is burr hole evacuation [3]. It can be performed under GA or MAC with scalp block. LA with sedation is a safe and efficacious technique when compared to GA in CSDH surgery [4]. However, the patient may need conversion to GA intraoperatively, for which adequate preparation has to be made prior. As these patients cannot respond to the effect of scalp block, change in vitals may be the criteria to check the effectiveness of the block. The patient needs to be carefully placed in the bed due to spasticity. In the case of MAC with sedation and without invasive ventilation, prefer an upper position (as far as possible depending on surgery) as it might support the patient’s respiration. Vessel cannulation, if difficult, might need an ultrasound or vein finder.

Special concerns with GA in ALS include risk of aspiration, enhanced sensitivity to non-depolarizing muscle relaxants (NDMRs), risk of rhabdomyolysis and hyperkalemia with depolarizing agents, and prolonged mechanical ventilation [1,6]. There is upregulation of nicotinic α7 acetylcholine receptors due to upper and LMN injuries or denervation. This leads to a larger potassium efflux into the bloodstream [7]. Rhabdomyolysis occurs when damaged sarcomeres release a large amount of dysfunctional proteins and electrolytes into circulation, which can cause severe injuries to the heart and kidneys [8]. Hence, the use of succinylcholine can lead to rhabdomyolysis and severe hyperkalemia, leading to cardiac dysrhythmias and death. In the case of NDMRs, short-acting drugs should be chosen to avoid prolonged effects and permanent motor neuron damage. They should be used with reversal agents to ensure quick recovery from muscle blockade [9]. Neuromuscular monitoring is indispensable when muscle relaxants are used as a part of GA. However, a discrepancy must be considered between measured neuromuscular response and clinical symptoms. A train-of-four stimulation (TOF) > 0.9 may not be used as the absolute criteria for safe extubation and full recovery from muscle paralysis in patients with ALS [10]. Additional monitoring, like bispectral index (BIS) (depth of anesthesia monitoring), arterial blood gas, and arterial line, may be used depending on disease severity. Opioids may worsen muscle rigidity, cause postoperative respiratory dysfunction, and must be used with caution. If required, short-acting opioids must be used. Weighing risks and benefits, general anesthesia without the use of any muscle relaxants should be considered [11]. Total intravenous (TIVA) or balanced anesthesia using volatile anesthetics appears safe. Among inhalational anesthesia, desflurane and sevoflurane should be preferred for maintenance due to their low lipid solubility, allowing for rapid reversal and dose adjustment [10]. Failure of the effect of the block may require conversion to GA. If GA is required, the patient can be induced with propofol and fentanyl, and an appropriate size of LMA can be inserted. Anesthesia should be maintained using oxygen, nitrous, and inhalational agents (preferably sevoflurane and desflurane) on spontaneous ventilation.

Lignocaine infusion can be used as it has been found to reduce opioid requirements [9]. Patients are susceptible to the possible neurotoxic effects of local anesthetics because of widespread demyelination. Hence, a calculated dose of local anesthetic must be administered [12]. Overall, regional anesthesia appears to be safe over GA in these patients.

Postoperative care needs to be tailored to individual patients' diseases, types of surgery, and anesthesia. Postoperative analgesia is an essential part of the care, as pain can lead to postoperative respiratory depression. Opioids should be avoided as they lead to respiratory depression; hence, non-steroidal anti-inflammatory drugs can be considered. Routine use of postoperative oxygen is not recommended because ALS patients have an inherent instability of respiratory control, and their drive for respirations when sleeping is based on oxygen saturation [13]. However, a stay in an intermediate or intensive care unit might be reasonable, as these patients are prone to postoperative respiratory failure, aspiration pneumonia, electrolyte abnormalities, hypovolemia due to poor nutrition intake, and exacerbation of neurologic symptoms and functional decline after surgery [14].

## Conclusions

Surgery may be necessary in patients with middle-stage ALS due to any concomitant problems. The anesthesia technique must be tailored to ensure the safe conduct of anesthesia and minimum impact on disease progression. It should also provide optimum operating conditions and adequate analgesia. Peripheral blocks or TIVA with inhalational agents are the preferred choice of anesthesia. GA with muscle relaxants and central neuraxial blocks should be avoided.
